# Implementing SARS-CoV-2 Rapid Antigen Testing in the Emergency Ward of a Swiss University Hospital: The INCREASE Study

**DOI:** 10.3390/microorganisms9040798

**Published:** 2021-04-10

**Authors:** Giorgia Caruana, Antony Croxatto, Eleftheria Kampouri, Antonios Kritikos, Onya Opota, Maryline Foerster, René Brouillet, Laurence Senn, Reto Lienhard, Adrian Egli, Giuseppe Pantaleo, Pierre-Nicolas Carron, Gilbert Greub

**Affiliations:** 1 Institute of Microbiology, Lausanne University Hospital and University of Lausanne, 1011 Lausanne, Switzerland; Giorgia.Caruana@chuv.ch (G.C.); Antony.Croxatto@chuv.ch (A.C.); Antonios.Kritikos@chuv.ch (A.K.); Onya.Opota@chuv.ch (O.O.); Rene.Brouillet@chuv.ch (R.B.); 2 Service of Hospital Preventive Medicine, Lausanne University Hospital and University of Lausanne, 1011 Lausanne, Switzerland; Eleftheria-Evdokia.Kampouri@chuv.ch (E.K.); Laurence.Senn@chuv.ch (L.S.); 3 Service of Infectious Diseases, Lausanne University Hospital and University of Lausanne, 1011 Lausanne, Switzerland; 4 Emergency Department, Lausanne University Hospital, 1011 Lausanne, Switzerland; Maryline.Foerster-Pidoux@chuv.ch (M.F.); Pierre-Nicolas.Carron@chuv.ch (P.-N.C.); 5 ADMed Microbiologie Laboratory, 2300 La Chaux-de-Fonds, Switzerland; Reto.Lienhard@ne.ch; 6 Clinical Bacteriology and Mycology, University Hospital Basel, 4001 Basel, Switzerland; Adrian.Egli@usb.ch; 7 Applied Microbiology Research, Department of Biomedicine, University of Basel, 4031 Basel, Switzerland; 8 Institute of immunology, University Hospital of Lausanne, 1011 Lausanne, Switzerland; Giuseppe.Pantaleo@chuv.ch

**Keywords:** SARS-CoV-2, COVID-19 diagnostic testing, rapid antigen test, health plan implementation, nucleocapsid protein, emergency ward

## Abstract

Following the Swiss Federal Office of Public Health (FOPH) authorization of the rapid antigen test (RAT), we implemented the use of the RAT in the emergency ward of our university hospital for patients’ cohorting. RAT triaging in association with RT-PCR allowed us to promptly isolate positive patients and save resources. Among 532 patients, overall sensitivities were 48.3% for Exdia and 41.2% for Standard Q^®^, Panbio^TM^ and BD Veritor™. All RATs exhibited specificity above 99%. Sensitivity increased to 74.6%, 66.2%, 66.2% and 64.8% for Exdia, Standard Q^®^, Panbio^TM^ and BD Veritor™, respectively, for viral loads above 10^5^ copies/mL, to 100%, 97.8%, 96.6% and 95.6% for viral loads above 10^6^ copies/mL and 100% for viral loads above 10^7^ copies/mL. Sensitivity was significantly higher for patients with symptoms onset within four days (74.3%, 69.2%, 69.2% and 64%, respectively) versus patients with the evolution of symptoms longer than four days (36.8%, 21.1%, 21.1% and 23.7%, respectively). Among COVID-19 asymptomatic patients, sensitivity was 33%. All Immunoglobulin-A-positive patients resulted negative for RAT. The RAT might represent a useful resource in selected clinical settings as a complementary tool in RT-PCR for rapid patient triaging, but the lower sensitivity, especially in late presenters and COVID-19 asymptomatic subjects, must be taken into account.

## 1. Introduction

Since the beginning and throughout the COVID-19 pandemic [1,2], SARS-CoV-2 has forced laboratories to constantly increase the number of tests performed and shorten the time to results.

In November 2020, the City of Lausanne (Vaud, Switzerland) had one of the highest SARS-CoV-2-positive rates worldwide, with about 1800 positive new cases per 100,000 inhabitants in 14 days [3]. During this time, Lausanne University Hospital (CHUV), a tertiary-care center of 1500 beds, experienced a massive influx of patients, rapidly exceeding its overall capacity, with up to 280 patients hospitalized for COVID-19, including 56 patients requiring intensive-care-unit (ICU) management (largely above the 30 ICU beds generally available).

Among patients consulting the emergency department, approximately 50% (around 20 patients per day) presented symptoms compatible with acute COVID-19, thus necessitating preliminary rapid triaging to facilitate their timely isolation.

From November 2, based on the Swiss Society of Microbiology recommendations [4], the Swiss Federal Office of Public Health (FOPH) authorized the use of rapid antigen tests (RATs) in addition to the gold-standard real-time reverse transcription-polymerase chain reaction (RT-PCR) [5]. According to these, the use of the RAT is indicated for (i) patients with acute respiratory symptoms for less than four days, not requiring hospitalization, or (ii) early triage of patients to be hospitalized, in the context of epidemiological emergencies (such as an outbreak), with a high number of patients admitted per day and a pretest probability above 20% [4].

Among the multiple antigen tests commercially available [6], the chosen reference standard antigen tests were the Standard Q^®^ COVID-19 Rapid Antigen Test (SD Biosensor, Korea/Roche, Switzerland) and the Panbio^TM^ COVID-19 Ag Rapid Test (Abbott, USA). These were validated in two Swiss clinical studies [7,8]. Both antigen tests showed high performances (sensitivity: 85% and 87.4%; specificity: above 99%) among patients with recent infection (less than seven days of symptoms) consulting outpatient testing centers in Geneva and Lausanne.

On 7 November, 2020, we implemented the diagnostic flow of SARS-CoV-2 infection at our hospital with a new RAT laboratory enforced in the heart of the emergency department to reduce delivery time, thus achieving early placement of positive patients in COVID-19-dedicated units and a reduction of rapid RT-PCR assays.

During this implementation, we compared the performances of the reference tests with two other RATs. These were chosen for their advantage to be fully automated, thus reducing the turnaround time (TAT) and human error rate. The performances of all tests were stratified according to different cycle thresholds (Cts)/viral loads (VLs) and the delay since symptom onset.

Furthermore, because of the characteristics of the patients admitted to the hospital, usually more severely ill or with a longer duration of illness, we hypothesized that the nasopharyngeal IgA might mask the antigen targets of RATs [9]. Several data in the literature already demonstrated the early production of IgA in response to SARS-CoV-2 infection, starting from the first week after infection [10,11]. Therefore, we measured nasopharyngeal IgA and compared the median Ct values between IgA-positive and IgA-negative patients.

## 2. Materials and Methods

### 2.1. Laboratory Set-Up and Testing Procedures

A RAT laboratory was built inside the emergency department, with two laboratory technicians dedicated to working there 8 h per day (from 9:00 to 18:30, given the highest activity during these hours), 7/7 days, receiving nasopharyngeal samples from patients consulting the emergency department.

**Figure 1:**
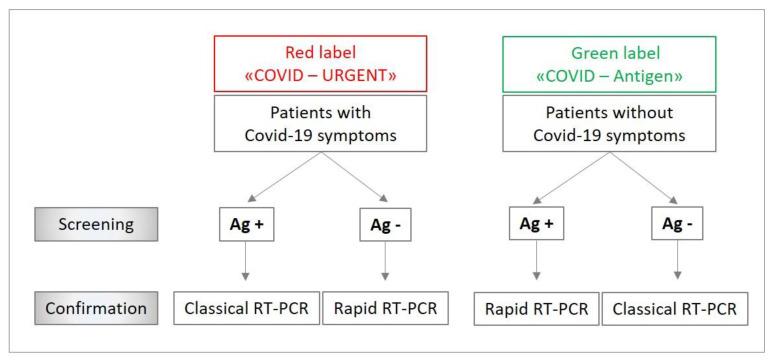
Diagnostic algorithm for managing tests flow according COVID-19 symptoms. Ag +: positive rapid antigen test. Ag −: negative rapid antigen test. RT-PCR: real-time polymerase chain reaction.

The samples were delivered from the patient to the RAT laboratory immediately after the sampling procedure and their labeling with the patient’s demographic details, hospital code and a red (COVID-19 symptomatic) or green (COVID-19 asymptomatic) label to quickly cohort patients according to our diagnostic algorithm (Figure 1).

**Figure 2:**
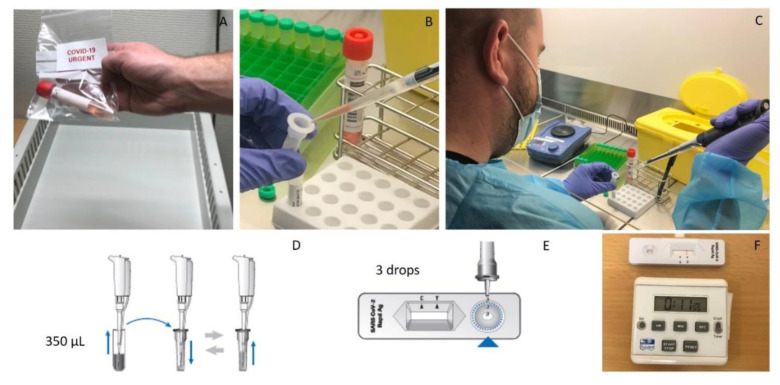
Antigen testing procedure with the reference test (Standard Q^®^). (**A**) Fresh nasopharyngeal sample from a COVID-19 symptomatic patient received at the RAT laboratory. (**B**–**C**) A 350 µL volume of the sample is collected from the viral transport medium and (**D**) mixed with the extraction buffer, according to the manufacturer’s instructions. (**E**) Three drops of the extracted sample are applied to the testing device. (**F**) Results are readable in 15 to 30 min.

Nasopharyngeal swabs were analyzed using Standard Q^®^, Panbio^TM^, Exdia COVID-19 Ag (Precision Biosensor Inc., Daejeon, Korea) and the SARS-CoV-2 BD Veritor™ System (Becton Dickinson, Franklin Lake, NJ, USA). Standard Q^®^ was considered the internal reference for RATs, and its results were used for patients’ care. With the purpose of performing RT-PCR confirmation on every sample while avoiding patients’ discomfort due to double sampling, the evaluation was done using a wet swab procedure, by suspending the nasopharyngeal swabs in 2.5 to 3 mL of viral transport media (VTM) solution. Then, 300 µL (for Panbio^TM^, BD Veritor™ and Exdia Immunoassay) or 350 µL (for Standard Q^®^) of the sample were mixed with the buffer solution and then tested, according to the manufacturer’s instructions (Figure 2).

The incubation and reading of the BD Veritor™ and Exdia tests were performed automatically with the provided reader after 20 min, and the reading of the Standard Q^®^ and Panbio^TM^ tests was carried out visually by the laboratory technician after 15 to 30 min of incubation.

### 2.2. Routine Confirmation by RT-PCR

All antigen results were confirmed on one of the following molecular platforms: (i) VIASURE SARS-CoV-2 (N1 + N2) Real-Time PCR Detection Kit for BD MAX™ (Becton Dickinson, Franklin Lake, NJ, USA) or GeneXpert^®^ SARS-CoV-2 test (Cepheid, Sunnyvale, CA, USA) [12] as rapid systems; (ii) test cobas 6800^®^ SARS-CoV-2 (Roche, Basel, Switzerland) [13] or our automated high-throughput Magnapure diagnostic (MDx) platform as classic systems [14,15]. The RT-PCR system (rapid versus classical) was chosen according to a diagnostic algorithm (Figure 1).

### 2.3. Study Design of the RAT Comparison

All patients admitted to the hospital wards from the emergency department, with or without COVID-19 symptoms, were systematically screened for SARS-CoV-2 and included in the study. Patients’ enrollment was stopped once we reached a collection of 100 PCR-positive samples and at least 200 PCR-negative samples, according to the SSM recommendations [4].

Clinical history, demographic details and nasopharyngeal specimens were collected for each patient as part of the standard of care. Microbiological data and time to results were extracted from our laboratory interface system (LIS). 

### 2.4. Statistical Analysis

To quantify the VL based on the number of Cts obtained with different molecular platforms, we used the following equation: VL = (10^((Ct − 40.856)/−3.697))*100. Details on the methods used to derive this equation were described elsewhere [16]. Bland–Altman analyses demonstrated a proportional bias in the intervariability of Ct measures between BD MAX™ and GeneXpert^®^ instruments compared to cobas 6800^®^ (see web-only Supplementary Table S1). To adjust for this bias, Passing–Balok regression equations were used to readapt the BD MAX™ (Ct calculated = 0.8829*Ct + 4.882) and GeneXpert^®^ Ct values (Ct calculated = 4.38 + 0.9*Ct if Ct > 20, or 3.85 + 0.92*Ct if Ct < 20) (see web-only Supplementary Figure S1).

We evaluated the positive and negative predicted values (PPVs, NPVs), sensitivity and specificity of each test with 95% confidence intervals (CIs) using an exact binomial test and a one-sided test. In each case, the overall accuracy and Kappa statistics were calculated. RT-PCR was used as a reference for sensitivity and specificity calculations. Analyses were stratified by VL categories and by the time delay of symptoms onset. Both Ct and different time delay categories were chosen based on recently published data in the literature [8,17].

A Wilcoxon rank-sum test with continuity correction and a Chi-squared test were performed to compare continuous and categorical variables, respectively, when appropriate.

Data were analyzed using “R statistical software” (version 3.6.1, 2019, Vienna, Austria).

### 2.5. Ethical Declaration

This article was prepared according to STANDARD guidelines for diagnostic accuracy studies reporting. The data on the viability of the different antigen assays were obtained during a quality enhancement project at our institution (CHUV, Lausanne, Switzerland). According to national law (Swiss Federal Act on Human Research), the performance and publishing of the results of such a project can be done without asking the permission of the competent research ethics committee.

This article was submitted to an online preprint archive [18].

## 3. Results

### 3.1. Emergency Department Patients Flow

**Figure 3:**
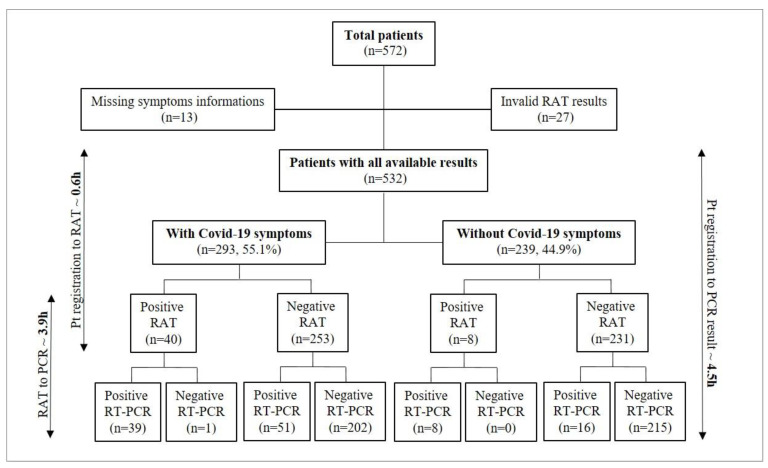
Number of patients included and time to results from patients’ registration to RAT or RT-PCR results. RAT: rapid antigen test. RT-PCR: reverse transcription-polymerase chain reaction. Pt: patients. H: hours. Please, note that the time-to-result analysis was performed on available results from 375 patients.

From November 7 to December 16, all patients presenting at the emergency department, with or without COVID-19 symptoms, were screened with RAT at the laboratory built inside the emergency ward. The Standard Q^®^ antigen test from Roche was used as a reference; thus, only its result was available to patients and clinicians. A total of 572 patients were screened. Among 532 patients with available results, 293 (55.1%) had symptoms consistent with COVID-19 and 239 (44.9%) were admitted for other reasons than COVID-19 suspicion (Figure 3).

Patients with COVID-19 symptoms were significantly older (*p* < 0.001), with a median age of 75 [IQR: 61–85] (Table 1).

No significant differences were found between symptomatic men and women (Table 1). As expected, there was a significant difference (*p* < 0.001) between the proportion of positive RAT results between symptomatic (13.7%) and asymptomatic (3.3%) patients, corroborated by RT-PCR results (30.7% versus 10%), while no significant differences were found between the median Cts of symptomatic versus nonsymptomatic patients (Table 1).

According to our diagnostic algorithm (Figure 1), patients with symptoms of COVID-19 tested positive with RAT and patients without symptoms of COVID-19 tested negative with RAT were confirmed with one of our classical RT-PCR platforms. The remaining patients, showing discordant results compared to their clinical presentation, were confirmed using one of our rapid RT-PCR platforms. All symptomatic patients with negative antigen results stayed at the emergency department in an isolation room or were admitted in a dedicated isolation unit until the result of RT-PCR. Overall, this strategy allowed us to save 271 rapid RT-PCR tests.

Among all patients submitted to the RAT, we documented one false-positive result and 67 false-negative results (51 among COVID-19 symptomatic patients and 16 among COVID-19 asymptomatic patients, as shown in Figure 3). The false-positive result belonged to a 61-year old patient who, because of the concordance between clinical presentation (suspicion of SARS-CoV-2 infection) and the result of the RAT, was cohorted in a room with other COVID-19-positive patients. Thanks to RT-PCR confirmation, we were able to identify the discordance and move the patient to another room as soon as the molecular results were available. To exclude possible cross-reactions of the RAT with other respiratory pathogens, a multiplex PCR panel (testing *Chlamydia pneumoniae*, *Mycoplasma pneumoniae*, adenovirus, parainfluenza virus 1 to 4, human metapneumovirus, pan-entero/rhinovirus, coronavirus E229, OC43, HKU1, NL63) was performed, which resulted negative.

### 3.2. Diagnostic Test Performance of RATs

A total of 572 patients were consecutively tested with the four RAT systems: Exdia, Standard Q^®^, Panbio^TM^, and BD Veritor™. All RATs were confirmed with one of the molecular platforms. Among 532 valid results, overall sensitivities varied from 48% to 41% among all tests, with PPV between 97% and 96%, NPV between 87% and 86% and an overall specificity greater than 99% for all RATs (Table 2).

**Figure 4:**
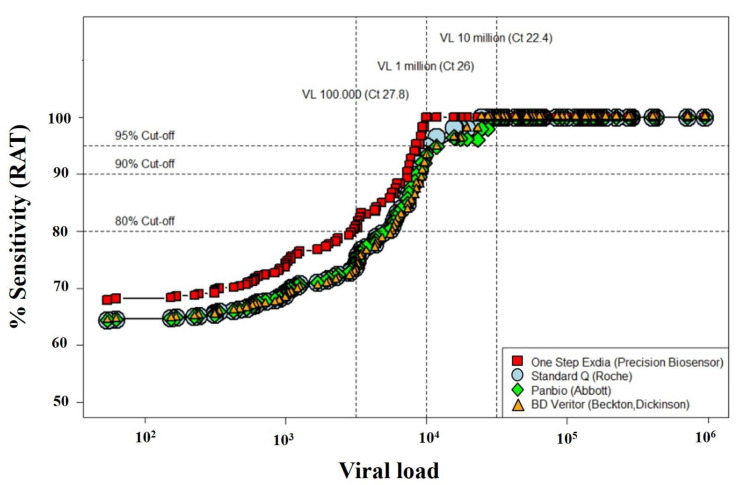
Comparison between four rapid antigen tests, showing the sensitivity according to the viral load. RAT: rapid antigen test. Viral load (VL) on the *x*-axis is expressed in logarithmic (log_10_) scale. Please note that the Exdia test exhibited the best performances, with 75.9% sensitivity for VL > 10^5^ copies/mL and 100% sensitivity for VL > 10^6^ and 10^7^ copies/mL.

Sensitivity rates increased to above 64% (74% for Exdia) for VLs greater than 10^5^, above 95% for VLs greater than 10^6^ and 100% for VLs greater than 10^7^ (Figure 4, see web-only Supplementary Table S2).

Subgroup analyses highlighted sensitivity rates below 34% among patients without COVID-19 symptoms, in contrast with symptomatic ones (Table 3).

**Figure 5:**
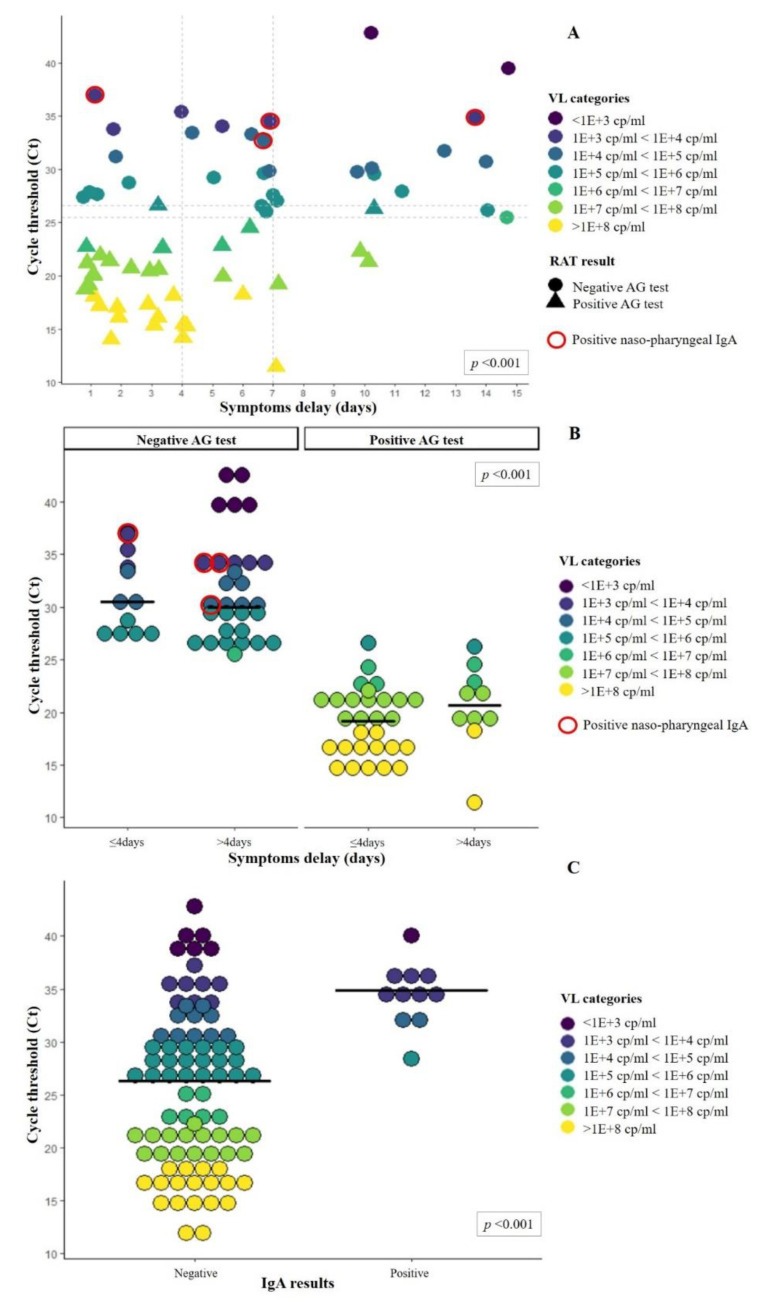
Cycle thresholds and viral loads of patients according to duration of symptoms and presence or absence of nasopharyngeal IgA.

Variations in overall sensitivity were also observed when accounting for patients’ duration of symptoms (Figure 5A–B and Table 3), with rates between 74% and 64% for patients with symptom delay shorter than four days versus rates between 36% and 21% for a delay longer than four days (Table 3).

The test results represented here are for the Standard Q^®^. Filled circles and triangles represent, respectively, negative and positive AG tests. Red circles represent samples tested positive for nasopharyngeal IgA. Colors represent different viral loads. (A) Viral loads distributions according to the duration of symptoms. Vertical dotted lines fall on Day 4 and 7 to highlight the number of positive tests after these 2 time points. Horizontal dotted line corresponds to 26.6 and 25.5 Ct, corresponding to the highest positive and lowest negative AG test, respectively. Median Ct was compared between patients with a symptom duration shorter or longer than 4 days (*p* < 0.01). (B) Viral load distributions and median Ct (solid line) among positive and negative antigen tests according to symptom duration. Comparison of median Ct between negative and positive RAT was statistically significant (*p* < 0.01). (C) Viral load distributions and median Ct (solid line) according to the nasopharyngeal IgA results. Please note that none of the 10 patients with positive IgA results had a positive RAT. Comparison of median Ct between negative and positive IgA was statistically significant (*p* < 0.01).

To further investigate the correlation of symptom duration and variations in VL, we also assessed these variables among patients admitted at CHUV from January to June 2020, during the first wave of SARS-CoV-2 infection. Data gathered from 444 patients showed a progressive reduction in VL over time, with median Ct starting around 22 for the first 4 days since the symptoms’ onset and significantly rising (*p* < 0.01) up to 32 when considering symptoms dated longer than 7 days (see web-only Supplementary Figure S2). 

Notably, when compared to RT-PCR, RAT underestimated the prevalence of 50% (Table 2). 

### 3.3. Time to Results Evaluation

We finally performed an assessment of the time to results between (i) the patient’s LIS registration, (ii) RAT results and (iii) RT-PCR confirmation (final diagnosis). Among 375 patients for whom time to results was available, a mean of 0.6 h (SD ± 1.8) since the time of patients’ registration was needed to obtain the result of the RAT, compared to a mean of 4.5 h (SD ± 6.4) for RT-PCR result. A mean delay of 3.9 h (SD ± 6.8) was observed between RAT and RT-PCR results (Figure 3).

### 3.4. Nasopharyngeal IgA

We tested 95 patients for nasopharyngeal IgA. Among them, 11/95 (11.6%) showed positive IgA levels (6 COVID-19 symptomatic and 5 COVID-19 nonsymptomatic patients), and all of them were tested negative for RAT. Conversely, all 41 patients who tested positive with RAT resulted negative for nasopharyngeal IgA, suggesting a possible implication of IgA in the competition with the antibodies used in the antigen assay. Remarkably, patients with nasopharyngeal IgA exhibited significantly higher median Cts (34.9, IQR 33.2–35.6) compared to those without IgA (26.3, IQR 19.2–30.7, *p* < 0.001, Figure 5C), thus further contributing to false-negative RATs.

## 4. Discussion

In this study, we described how to implement and use RAT in an emergency room. Overall, we were able to identify and isolate COVID-19-symptomatic and RAT-positive patients within an average of 40 min from their registration, also saving around 50% of the reagents for rapid RT-PCR during this period. This management proved to be a valuable resourcein the context of rapid molecular reagents shortage. The short time to results might also have played a pivotal role in the early placement of SARS-CoV-2-positive patients into COVID-19 units, thus reducing risks of cross-transmission in the emergency department. 

We also carried out a performance assessment and comparison between four RATs; because of the best performances of Exdia and the more convenient automatized reading method, this test was adopted as a reference at our hospital.

**Figure 6:**
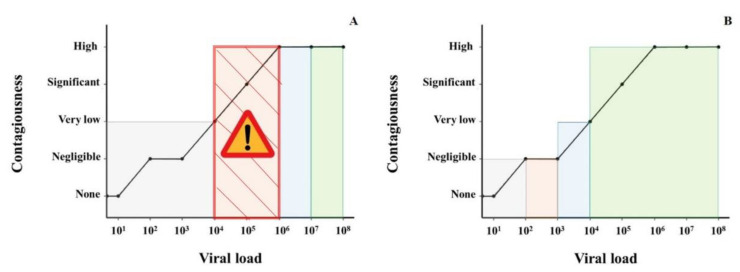
Relationship between viral load, hypothetical contagiousness and correspondence with the sensitivity of (**A**) RAT and (**B**) RT-PCR (based on our evaluation).Viral load (VL) on the *x*-axis is expressed in a logarithmic (log_10_) scale. The gray area represents a test sensitivity of <20%. The orange area represents a sensitivity of >20% but lower than 80%. The blue area represents a sensitivity of >80% but <95%. The green area represents a sensitivity above 95%. (**A**) The “Attention” symbol highlights the orange dashed critical zone where SARS-CoV-2 is already contagious but the sensitivity of the RAT is not acceptable, being very low (30–60%) for VL between 10^4^ and 10^5^ and low (60–80%) for VL between 10^5^ and 10^6^.Contagiousness is estimated to be negligible when R0 < 0.01, very low when R0 > 0.01 and <0.05, significant when R0 > 0.05 and < 0.2 and high when R0 > 0.2 over a 24 h period in the same room than another patient.

Considering an acceptable sensitivity rate above 80%, none of the antigen tests reached that threshold among patients with viral loads below 10^6^ copies/mL, and sensitivity rates became good and excellent with VL above 10^6^ copies/mL (Figure 6).

Data from a recent epidemiological study on SARS-CoV-2 epidemics in Switzerland [19] demonstrated the possible onset of clusters of infections originating from patients with VLs below 10^5^ copies/mL. This indirectly highlights the risk associated with a massive (only) RAT screening, which would likely lead to much larger cases of clusters in the population when the first cases are missed by using RATs.

When considering lower sensitivities for patients with symptoms delays greater than four days (Table 3), we explained that with lower VLs associated with that time span. Indeed, the results from the VL time-varying assessment among patients admitted to CHUV during the first wave of infections corroborated this hypothesis, showing a progressive reduction in VL over time. The very low sensitivity (<32%, Table 3) of antigen tests in subjects with symptoms for more than seven days is likely due to the low viral load generally observed after a week of disease in immunocompetent subjects (see web-only Supplementary Figure S2). Hence, our results corroborated the importance of following official FOPH recommendations, limiting RAT screening within the first four days of symptoms [4,5].

Notwithstanding the enthusiasm for a rapid, cheaper and easy diagnostic solution such as antigen tests, the comparison between RAT and RT-PCR sensitivity (Figure 6) still highlights important differences in detection rates. It becomes clear that if we had only used RAT, not only would the prevalence of the disease have been underestimated, but also a significant number of patients with a high VL (from 10^4^ to 10^6^ copies/mL and thus potentially contagious) would not have been identified and therefore not put on isolation measures. This is even more relevant among hospitalized patients (with the majority of two to five bedrooms) with important comorbidities (hence more likely to develop complications), where appropriate cohorting would mean that fewer, potentially susceptible patients would be exposed for a prolonged duration to SARS-CoV-2.

We believe that during epidemic waves, antigen tests may also prove to be useful at hospitals’ emergency rooms for patients’ cohorting, especially when rapid RT-PCR reagents are not available in sufficient numbers due to reagent shortage and provided that all RATs are confirmed by a PCR.

For outpatients testing, RAT can still represent a useful resource in the context of massive screening, limiting such assays to subjects with less than four days of symptoms and not considered vulnerable. 

To compensate for the lower sensitivity of the RAT, the testing frequency could be increased, with the purpose of catching a greater number of patients during their high VL infection phase [20]. Nevertheless, RAT cannot substitute but only complement the RT-PCR testing of outpatients, because of the gap in detection left among those patients with a VL, high enough to be contagious but not high enough to be detected by antigen assays.

Current literature shows discordant results on the sensitivity rates of the RAT. In line with recent reports [1,2,21,22], our results showed a significantly lower overall detection rate of SARS-CoV-2 infections compared to the previous validation we performed in Lausanne and other studies [8,23,24]. First, this is due to the different patient populations admitted to the emergency ward of our hospital compared to outpatients (lower VL among hospitalized subjects). Second, the study performed in the outpatients’ clinic was done at the time of the explosive growth in the number of cases, with most subjects exhibiting a recent infection. Third, patients arriving at the emergency ward were probably sicker, often for a longer time, and possibly already developed an immune response, leading more frequently to lung edema, radiological infiltrate and hypoxemia. The presence of mucosal IgA targeting SARS-CoV-2 surface antigens might have played a role, thus competing with RAT for the same target. Interestingly, in our study, none of the 41 patients with a positive RAT showed the presence of IgA, reinforcing the hypothesis that IgA might compete with the antibodies used in the antigen assay. Finally, we hypothesized that the dilution of the sample in the VTM might have affected the sensitivity. However, very low sensitivity of 28% (even lower than the 33% sensitivity observed in the present study) was also observed with the same Standard Q^®^ antigen test among subjects without COVID-19 symptoms, hospitalized in another hospital, despite their use of a dry swab approach (Caruana et al., submitted).

## 5. Conclusions

Given the huge gap in the sensitivity between RAT and RT-PCR (roughly corresponding to about 10,000-fold reduced analytical sensitivity), RAT could be cautiously used, especially as complementary to PCR-based screening, in nonvulnerable outpatient subjects with one to four days of symptoms, when the viral load is likely very high. 

During the peak of outbreaks, when patient flow to the emergency department is particularly high and early orientation and effective cohorting are crucial, RAT might be used in conjunction with RT-PCR, especially when the laboratories are facing a shortage of rapid RT-PCR assays. Overall, the RATs exhibit a sensitivity lower than 50%, which is even lower than flipping a coin. Therefore, in the near future, when enough rapid RT-PCR assays become available, the use of the RAT will be questionable, even to screen nonvulnerable symptomatic outpatient subjects.

## Figures and Tables

**Table 1:** Baseline characteristics and estimated disease prevalence according to different diagnostic approaches, in subjects with or without symptoms of COVID admitted at the emergency ward of Lausanne University Hospital. Please note that in this setting, the RAT only detected about one-third of asymptomatic hospitalized patients positive by RT-PCR.

	Without COVID-19 Symptoms (N = 239)	With COVID-19 Symptoms (N = 293)	*p*-Value
Gender			
Female	105 (43.9%)	131 (44.7%)	0.927
Male	134 (56.1%)	162 (55.3%)	
Age			
Median (IQR) years	67.0 [48.5, 81.0]	75.0 [61.0, 85.0]	<0.001
SARS-CoV-2 RT-PCR result			
Negative	215 (90.0%)	203 (69.3%)	<0.001
Positive	24 (10.0%)	90 (30.7%)	
SARS-CoV-2 RAT * result			
Negative	231 (96.7%)	253 (86.3%)	<0.001
Positive	8 (3.3%)	40 (13.7%)	
Cycle threshold			
Median (IQR)	29.8 [22.6, 35.1]	27.0 [20.5, 32.5]	0.274
Missing	215 (90.0%)	203 (69.3%)	

* RAT results represented here were obtained with the Standard Q^®^ test, which was used for patients’ care. RAT: rapid antigen detection. RT-PCR: real-time polymerase chain reaction. IQR: interquartile range.

**Table 2:** Estimated disease prevalence, sensitivity and specificity according to different tests.

Type of RAT	Prevalence with RT-PCR	Prevalence with RAT	Overall Sensitivity of RAT	Overall Specificity of RAT	RAT Accuracy [95% CI]
Exdia	114/532 (21.4%)	57/532 (10.7%)	48.3%	99.5%	88.5% [85.5–91.1]
Standard Q^®^	114/532 (21.4%)	48/532 (9%)	41.2%	99.7%	87.2% [84.1–89.9]
Panbio^TM^	114/532 (21.4%)	49/532 (9.2%)	41.2%	99.5%	87.0% [83.9–89.8]
BD Veritor^TM^	114/532 (21.4%)	48/532 (9%)	41.2%	99.7%	87.2% [84.1–89.9]

RAT: rapid antigen test. RT-PCR: real-time polymerase chain reaction.

**Table 3:** Overall sensitivity and specificity rates according to different tests, symptoms onset delay and compared between COVID-19 symptomatic and asymptomatic patients.

	Se./Sp. Rates (Without COVID-19 Symptoms)	Se./Sp. Rates (With COVID-19 Symptoms)			
RAT	Overall (n = 239)	Overall (n = 293) *	Symptoms Onset Delay ≤4 Days (n = 138)	Symptoms Onset Delay 4 ≤ 7 Days (n = 46)	Symptoms Onset Delay >7 Days (n = 44)
Exdia - Se. - Sp.	33% 100%	52.2% 99%	74.3% 100%	43.7% 96.7%	31.8% 95.4%
Standard Q^®^ - Se. - Sp.	33% 100%	43.3% 99.5%	69.2% 98.9%	25% 100%	18.2% 100%
Panbio^TM^ - Se. - Sp.	33% 99.5%	43.3% 99.5%	69.2% 98.9%	25% 100%	18.2% 100%
BD Veritor^TM^ - Se. - Sp.	33% 100%	43.3% 99.5%	64% 100%	37.5% 100%	13.6% 95.4%

* For 65/293 subjects labeled as COVID-19 symptomatic, either data were missing on symptoms onset delay or patients did not show typical COVID-19 symptoms (differential diagnosis was not in favor of COVID-19). RAT: rapid antigen test. Se.: sensitivity. Sp.: sensitivity.

## Data Availability

Data supporting reported results will be available upon request for the peer-review process.

## Supplementary Materials

The following are available online at https://www.mdpi.com/article/10.3390/microorganisms9040798/s1. Figure S1: Passing-Bablock regressions for cobas 6800^®^ Ct gene E against: A. GeneXpert^®^ gene E (for Ct > 20); B. GeneXpert^®^ gene E (for Ct < 20); C. BD MAX™ gene N2. Figure S2: Changes of SARS-CoV-2 viral load over time among patients admitted between January and June 2020 according to symptoms delay. Table S1: Bland–Altman analyses for inter-variability of Ct measure between cobas 6800^®^ (Roche) versus GeneXpert^®^ (Cepheid) or BD-Max^TM^ (Becton, Dickinson). Table S2: RAT sensitivity rates after stratification for viral load.
