# Supplementary material for: Implementing SARS-CoV-2 Rapid Antigen Testing in the Emergency Ward of a Swiss University Hospital: The INCREASE Study
Source: Microorganisms. 2021 Apr 10;9(4):798. doi: 10.3390/microorganisms9040798 (PMC8069749; doi:10.3390/microorganisms9040798)
Supplement: Supplementary file 1 [file microorganisms-09-00798-s001.pdf]

Figure S1: Passing-Bablock regressions for cobas 6800® Ct gene E against: A. GeneXpert® gene E (for Ct >20); B. GeneXpert® gene E (for Ct <20); C. BD-MAX™ gene N2.

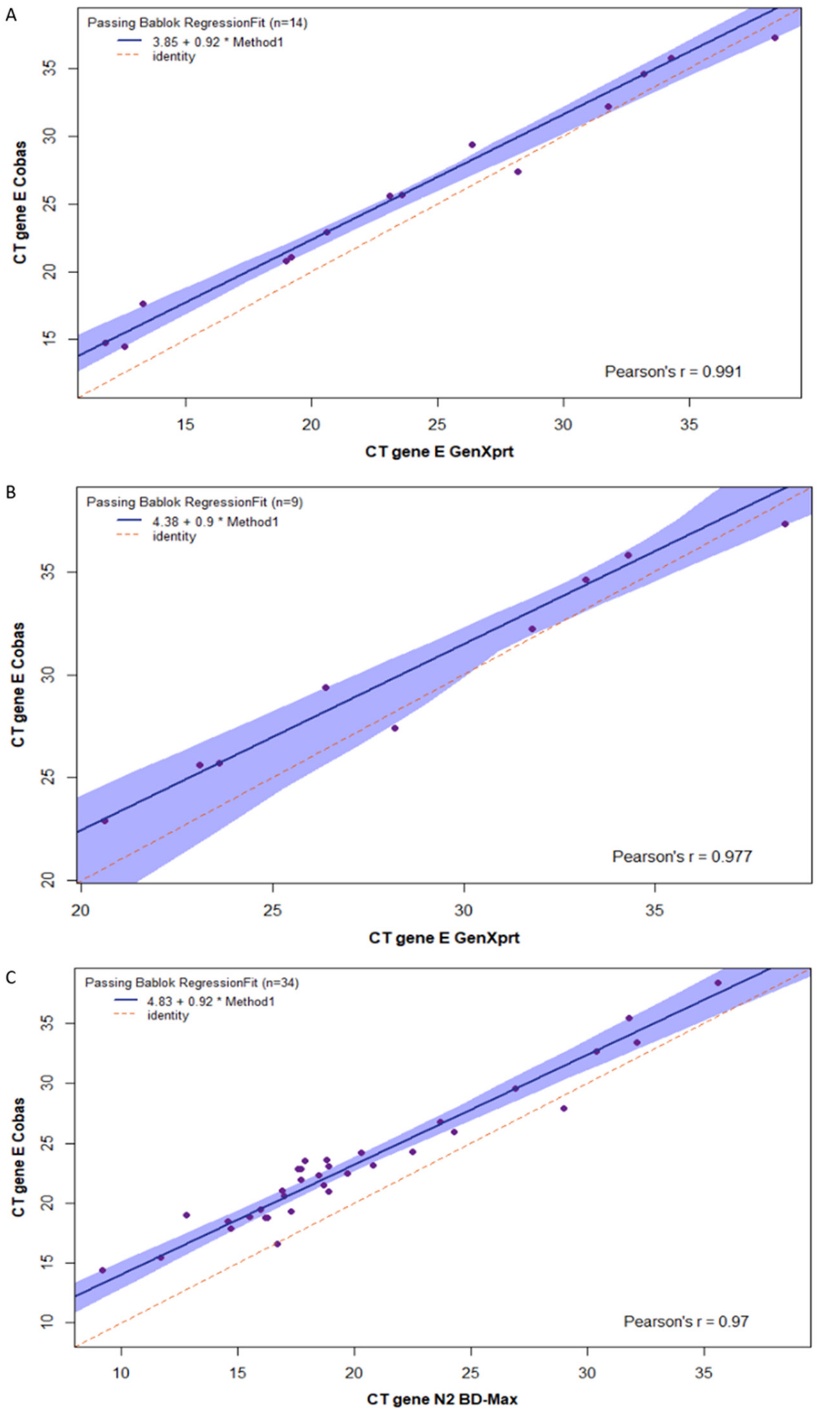

Method 1: Ct gene E cobas6800©

Figure S2: Changes of SARS-CoV-2 viral load over time among patients admitted between January and June 2020 according to symptoms delay.

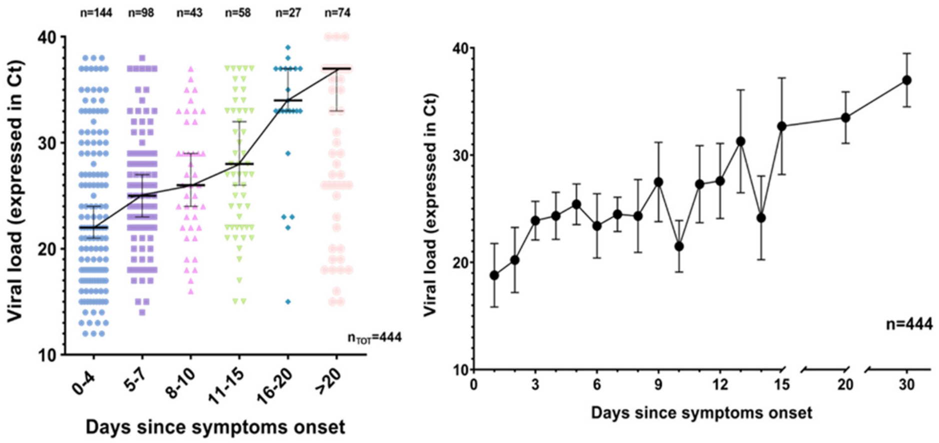

Table S1: Bland-Altman analyses for inter-variability of Ct measure between cobas 6800® (Roche) versus GeneXpert® (Cepheid) or BD-Max™ (Becton, Dickinson).

	**Proportional bias [95% CI]**	**Upper LoA [95% CI]**	**Lower LoA [95% CI]**	***p*-value**
Overall cobas vs GeneXpert (n=14)	-1.7 [-2.5;-0.9]	1.1 [-0.3;2.6]	-4.5 [-6.1;-3.0]	<0.001
Cobas vs GeneXpert for Ct >20 (n=9)	-1.2 [-2.4;-0.1]	1.6 [-2.5;-0.9]	-4.1 [-6.1;-3.1]	0.03
Overall cobas vs BD-MAX (n=34)	-3.2 [-3.7;-2.7]	-0.2 [-1.1;0.7]	-6.2 [-7.1;-5.3]	<0.001

Table S2: RAT sensitivity rates after stratification for viral load.

**RAT**	**Sensitivity among patients with VL>10^5 (n=71)**	**Sensitivity among patients with VL>10^6 (n=46)**	**Sensitivity among patients with VL>10^7 (n=38)**
Exdia	74.6%	100%	100%
Standard Q	66.2%	97.8%	100%
Panbio	66.2%	96.6%	100%
BD Veritor	64.8%	95.6%	100%
